# Myelinated retinal nerve fibers

**DOI:** 10.11604/pamj.2014.17.97.3144

**Published:** 2014-02-07

**Authors:** Hanan Handor, Rajae Daoudi

**Affiliations:** 1 Université Mohammed V Souissi, Service d'Ophtalmologie A de l'Hôpital des Spécialités, Centre Hospitalier Universitaire, Rabat, Morocco

**Keywords:** Myelinated, retinal nerve, exotropia

## Image in medicine

A 7-year-old girl of second degree consanguineous parents, presented with an 8 months history of intermittent exotropia of the left eye. Cycloplegic refraction was -0.25.-0.50×165° and -3.00.-0.50×20° in the right and left eyes respectively. An initial objective assessment of the visual function showed a best corrected visual acuity of 12/10 in the right eye and 6/10 in the left eye. Fundoscopy revealed superficial whitish, opaque lesion with feathered edges, hiding the optic disc and retinal vessels, and following the course of the nerve fiber layer in the left eye. We conclude to the diagnosis of myelinated retinal nerve fibers. General examination was unremarkable. The treatment of amblyopia was then undertaken, and a final corrected visual acuity of 9/10 was attained in the left eye. Myelinated retinal nerve fibers are a rare idiopathic developmental abnormality, occurring when the myelination of ganglions cells axons extend abnormally into the retina. Usually, this malformation does not affect visual acuity, unless it involves the fovea. Otherwise it can be, rarely, associated with myopia, strabismus, and amblyopia. Amblyopia treatment should be systematically considered, even if it's reported to be refractory to the treatment in such cases. General clinical assessment must be carried out in order to exclude some systemic disorders that may be linked to this abnormality including neurofibromatosis type 1, craniofacial abnormalities, skeletal malformations, and basal cell nevus syndrome.

**Figure 1:**
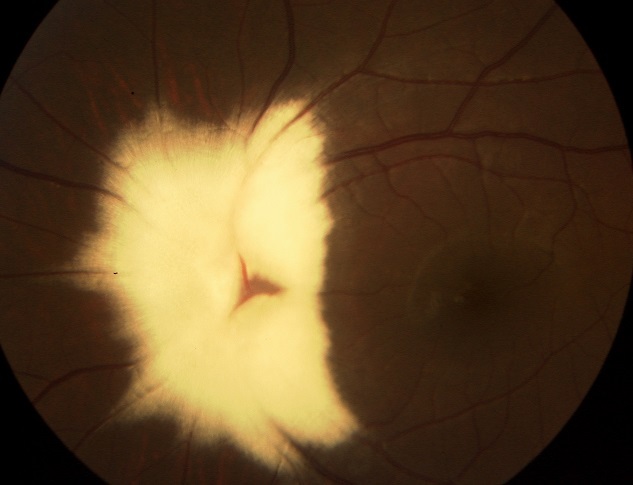
Funduscopy of the left eye revealing extensive whitish lesion involving the optic disc

